# From mechanisms of carcinogenesis to early intervention: an interview with Ashok Venkitaraman

**DOI:** 10.1242/dmm.052164

**Published:** 2024-12-23

**Authors:** Ashok Venkitaraman

**Affiliations:** ^1^ Cancer Science Institute of Singapore, National University of Singapore, Singapore 117599; ^2^ Institute of Molecular and Cell Biology, 61 Biopolis Drive, Singapore 138673


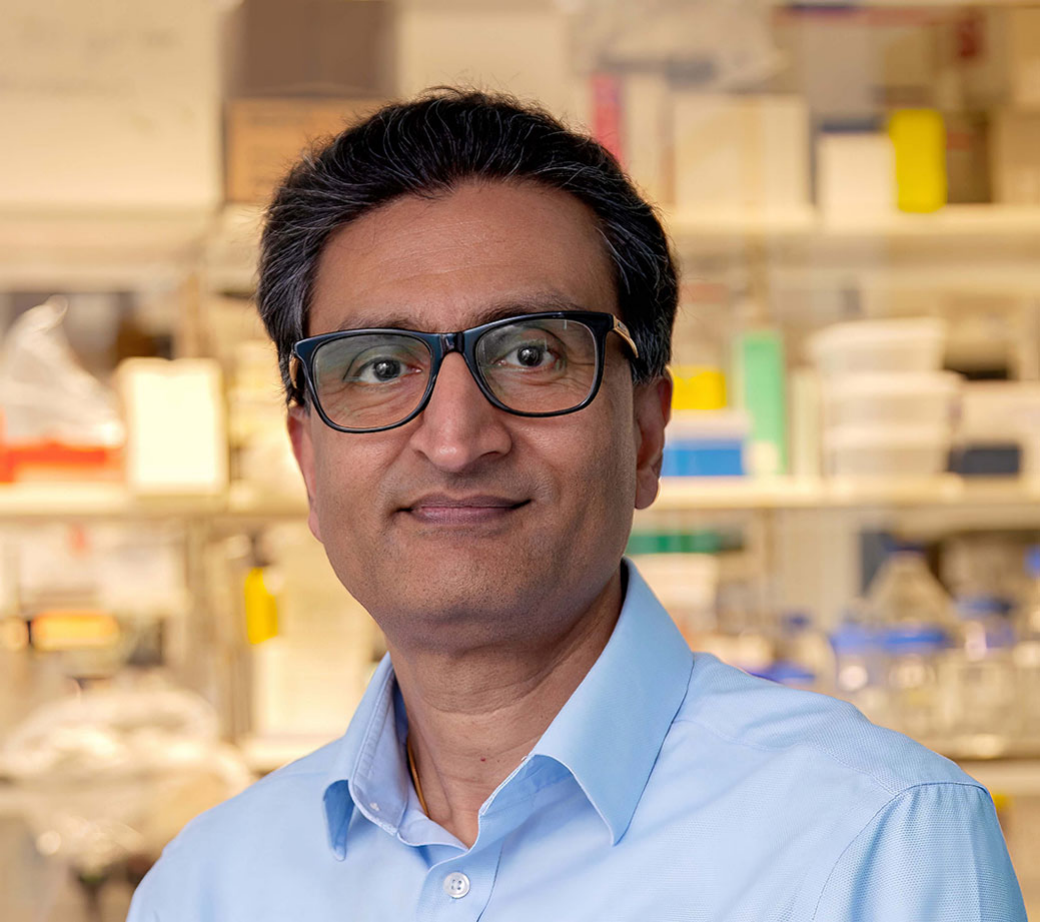


**Ashok Venkitaraman**

Ashok Venkitaraman has made seminal discoveries elucidating the tumour suppressive mechanisms that maintain genome integrity through his pivotal studies on the breast cancer gene *BRCA2*, thereby illuminating the role of a class of genes often inactivated in human cancers. Ashok initially studied medicine at the Christian Medical College, Vellore, India. He then moved into fundamental research by completing a PhD at University College London in Marc Feldman's laboratory. Ashok was first a group leader at the Medical Research Council (MRC) Laboratory of Molecular Biology (LMB) in Cambridge from 1991 to 1998. In 1998, he was elected as the first holder of the Ursula Zoellner Professorship at the University of Cambridge, and was later the Director of the MRC Cancer Unit and Joint Director of the Hutchison/MRC Research Centre from 2006 to 2019. In 2020, Ashok moved his laboratory to Singapore, where he is Director of the Cancer Science Institute of Singapore and Distinguished Professor of Medicine at the Yong Loo Lin School of Medicine, National University of Singapore. He is also Research Director in the Institute of Molecular and Cell Biology at the Agency for Science, Technology and Research (A*STAR). Owing to his prominent research achievements, Ashok has been elected a Fellow of the Academy of Medical Sciences and a Member of the European Molecular Biology Organization (EMBO). In 2017, he was awarded the Basser Global Prize for his groundbreaking research uncovering how BRCA2 suppresses cancer by protecting genome integrity. Ashok's work-advancing drug discovery has led to several spin-out companies, including PhoreMost and Sentinel Oncology. Here, we discuss his outstanding research journey, how this is translating to tangible clinical outcomes and how these outcomes can be shared globally.

**What made you decide to move into fundamental and translational research after studying medicine?**

I trained in a small medical school, in a small town in the south of India. Unusually – because the Christian Medical College ran an innovative training programme for its students that took us out into the local villages – this experience exposed me to what it's like to practice medicine in rural India. For me, that was a revelation. It led to the realisation that, in the face of such extreme need, often driven by socioeconomic circumstances, I could do little as a medical practitioner. Not surprisingly, I turned towards research, with the ambition that my work could impact more people than I would have ever been able to help as a clinician.

Anything I've achieved in my career was founded on the generosity and mentorship of many individuals. In India, when I was training at medical school, research was not part of the curriculum. At that time, Jacob John was the Professor of Virology at the Christian Medical College Hospital in Vellore. He pioneered community-based vaccination against diseases like polio, and I had the great fortune to meet him when I was an undergraduate. He allowed me to work in the virology laboratories at night and in the early mornings, outside my lectures. I learned how to ask scientific questions and define experiments, and was able to publish several papers on herpesvirus infections in India. Most importantly, this experience fired up my desire to focus seriously on research.

There are many, many people across the developing world who have the potential to contribute to science, but are never able to realise it, not because they lack talent, but because they lack opportunity. As one of the fortunate few, giving back what I can has motivated me for many years.

**Throughout your career you have continued to support research in India. Why is this an important endeavour to you?**

Put simply, ‘There but for the grace of God’, has run through my mind throughout my career. When I was working in India decades ago, it was not easy for someone like me, with no formal research training or a PhD at that stage, to find the right opportunities and environment to become a better researcher or to get access to cutting-edge technologies. I was helped by the generosity of Gilbert Lenoir, who at that time was the head of one of the divisions of the World Health Organization (WHO)’s International Agency for Research on Cancer in Lyon, in France. Gilbert helped me, while I was still a medical student in India, to get WHO funding, which allowed me to work in France for several months and in Bristol for a month or two, where I met inspirational researchers like Tony Epstein and Alan Rickinson. During this time, I was also able to visit Marc Feldman, a leading immunology researcher in London. After we met, Marc offered me the opportunity to do a PhD in his lab. So, Gilbert's generosity opened doors, which enabled me to better fulfil what potential I had. There are many, many people across the developing world who have the potential to contribute to science, but are never able to realise it, not because they lack talent, but because they lack opportunity. As one of the fortunate few, giving back what I can has motivated me for many years. More recently, my journey has taken me to Singapore, a highly developed nation at the heart of Asia, which I believe has the talent, infrastructure and potential to be a beacon for biomedical research.

**You've made seminal discoveries in your career, but what discovery or research project have you found most exciting to work on?**

I'm afraid that I will have to give you more than one example! I have been excited by so many projects.

The first example comes from the problem I initially worked on in my independent lab at the MRC LMB in Cambridge, after my postdoctoral work with Michael Neuberger. The adaptive immune response is initiated by specific, yet diverse, antigen receptors seated on the surface of B and T lymphocytes. Antigen receptor diversification is driven at the DNA level by the rearrangement of V, D and J segments distributed across large genomic regions. How does the enzymatic machinery executing rearrangement access such extensive genomic landscapes? We discovered that a cytokine receptor, the interleukin (IL)-7 receptor, which has a critical role in early lymphopoiesis, transmitted a signal to ‘open up’ the accessibility of the antibody heavy chain gene locus to cutting and repair by the rearrangement enzymes, RAG1 and RAG2 (Corcoran et al., 1998). This mechanism was later shown by Mark Schlissel and others to work for T-cell receptor gene loci, too. Therefore, our work raised the idea that antibody diversity was influenced by extrinsic signals, independent of antigen, early during lymphocyte development. As the first significant discovery from my independent lab, it's been exciting to see later implications of this work in the recent use of IL-7 for the therapy of cancer and immunodeficiency syndromes.

Soon afterwards, our work on V, D and J segment rearrangement in the genome of immune cells took a wholly unexpected twist! I had been aware that the abnormal cutting and pasting of large genomic segments could generate the chromosomal instability that is near-universally prevalent in cancer cells from epithelial malignancies, but never thought that my work would switch towards understanding their genesis. What made the connection was a series of fortuitous events.

Around that time, the breast cancer genes *BRCA1* and *BRCA2* had just been cloned, through large transnational collaborations led by Mary-Claire King and Michael Stratton, to identify the genetic defect in women with a high risk of developing familial breast and ovarian cancer. There was emerging evidence from Alan Bradley, Alan Ashworth and Tak Mak to suggest that cells lacking BRCA2 were sensitive to sources of DNA damage, like X-rays.

Genes involved in V(D)J rearrangement in the immune system are known to be required for the repair of X-ray-induced DNA breaks. So, in our lab, I made the hypothesis that BRCA1 and BRCA2 might participate in double-strand DNA break repair and thereby be involved in V(D)J rearrangement. I approached Bruce Ponder and Martin Evans, who had created a *BRCA2* knockout murine strain, for material to test this idea. However, part of the idea – namely, that BRCA2 might regulate V(D)J rearrangement – was soon shot down when Ann Corcoran, then a postdoc in my lab, showed that lymphocytes from the knockout mice exhibited no such defect. Nevertheless, Ketan Patel, who was also then a postdoc in my lab, and Veronica Yu, then my PhD student, found that fibroblasts from the *BRCA2* knockout mice had growth and cell-cycle defects, plus a spectrum of sensitivities to DNA-break-inducing genotoxins, that together pointed to the involvement of BRCA2 in double-strand DNA break repair. Our work culminated in the demonstration that chromosomes from BRCA2-deficient cells spontaneously acquired breaks and formed abnormal structures termed ‘radial chromosomes’ when dividing in cell culture. Breaks affecting just one of the two sister chromatids, as well as tri-radial and quadri-radial chromosomes, were abundant, reflecting defects in mitotic recombination. Interestingly, these chromosomal lesions were quite similar to those that have been observed in other genetic diseases that predispose to cancer, such as Bloom syndrome and Fanconi anaemia. So, our findings collectively provided evidence to implicate *BRCA2* in homology-directed DNA recombination between mitotic chromosomes. They also suggested that the pathogenesis of BRCA2-deficient tumours might recapitulate features of carcinogenesis associated with Bloom syndrome and Fanconi anaemia. Of course, it's been very satisfying to see that both of those early predictions have turned out to be correct! *BRCA2* is indeed a central player in homology-directed DNA recombination (e.g. Moynahan et al., 2001), and our later work has gone on to help elucidate its mechanism (e.g. Pellegrini et al., 2002). Moreover, rare bi-allelic mutations in *BRCA2* can give rise to Fanconi anaemia (Howlett et al., 2002).

Despite our increasingly precise knowledge of how BRCA2 works at the molecular level, we still do not fully understand how patients carrying *BRCA2* mutations develop cancer. Given my medical training, I suppose it has been natural for my lab to gravitate towards this problem, in work that continues to this day. I'll cite one last example of this work, which, in retrospect, has perhaps been the most difficult and most exciting problem we've taken on.

Patients at a high risk from familial breast and ovarian cancer typically inherit one mutant and one normal (‘wild-type’) copy of *BRCA2*. As long as one wild-type copy of *BRCA2* remains, cells seem to be pretty normal in their capacity to carry out the cancer-preventing functions of BRCA2 that preserve genome stability. Indeed, Al Knudson in the 1970s had proposed that both copies of tumour-preventing genes like *BRCA2* must be inactivated to initiate carcinogenesis. This idea – called the Knudson ‘two-hit’ paradigm – has dominated contemporary thinking about the role of cancer-preventing genes, or ‘tumour suppressors’. Also, whether both copies of *BRCA2* are inactivated in cancers from mutation carriers is proving to be quite important because sensitivity to targeted therapies, like platinum compounds or poly-ADP ribose polymerase (PARP) inhibitors, only occurs when that second wild-type copy of *BRCA2* is lost. So, we developed the first model in the mouse germline that faithfully recapitulated features of *BRCA2*-dependent carcinogenesis. We chose to work on *BRCA2*-associated pancreatic cancer, not only because *BRCA2* mutation carriers have higher susceptibility to this disease, but also because we could leverage earlier work by Dave Tuveson and Tyler Jacks, in order to create a new model for familial pancreatic cancer in my lab.

Our first results were unexpected! Ferdinandos Skoulidis, at that time an MD, PhD student in my lab, working with Liam Cassidy, also then my PhD student, found that inactivation of a single copy of *BRCA2* was sufficient to accelerate pancreatic carcinogenesis in our new model…but the tumours retained an intact second copy, which was still expressed normally, and remained resistant to PARP inhibitors. To my later chagrin, I did not believe these early results could be correct, and asked Ferdinandos and Liam to back up their observations with increasingly detailed experiments on more and more tumour samples, all of which supported their initial findings. We were fortunate enough to get a few precious samples of pancreatic cancer tissue from the Icelandic Cancer Registry, from carriers of a founder mutation in *BRCA2* that is quite common in Iceland, and were able to validate our findings. So, *BRCA2*, at least in our model, can violate the Knudson two-hit paradigm (Skoulidis et al., 2010). Later data from The Cancer Genome Atlas project, and from Susan Domchek and Kate Nathanson's work, also supported this notion.

Our quest to understand what was going on has led us in exciting new directions. Individuals even in families carrying the same germline *BRCA2* mutation exhibit differences in their age of cancer onset and the type of cancers they may develop, speaking to the existence of modifiers of cancer risk. A few years ago, my lab identified a potential gene–environment interaction that could modify carcinogenesis in *BRCA2* mutation carriers. As I've said before, non-cancerous cells from *BRCA2* mutation carriers typically express around 50% of the normal levels of BRCA2 protein, because one of the two copies of the *BRCA2* gene is inactive. Nevertheless, this amount of BRCA2 protein seems to be sufficient for cancer-preventing functions. So, what makes these cells more susceptible to carcinogenesis? We found that when cells carrying a single copy of *BRCA2* are challenged with small concentrations of certain aldehydes, a pervasive class of chemical compounds, BRCA2 protein is quickly degraded by what our preliminary evidence suggests may be a ubiquitin-independent, but proteasome-dependent, pathway (Tan et al., 2017). When this happens, we found that these aldehyde-exposed cells temporarily lost the essential functions of BRCA2 in DNA repair. So, our findings raise the idea that aldehyde exposure – by temporarily inactivating BRCA2 – might push otherwise normal cells carrying a single copy of *BRCA2* towards carcinogenesis.

Aldehydes, like formaldehyde, are around everywhere in our environment, and they're also generated by many important metabolic processes within cells. In fact, energy metabolism changes early in carcinogenesis as nascent cancer cells switch from ATP generation by oxidative phosphorylation to ATP generation by glycolysis. This change, termed metabolic reprogramming, is considered a hallmark of cancer. In work published earlier this year (Kong et al., 2024), we have been able to show that an aldehyde byproduct of glycolysis, methylglyoxal, can inactivate BRCA2 by protein degradation, triggering genome-wide patterns of single-base substitution mutations that are implicated in cancer initiation. Interestingly, we found that pancreatic cancer cells from the murine model we created to first reveal that *BRCA2* could violate Knudson's two-hit paradigm also contain high levels of methylglyoxal, and sustain similar types of genome-wide mutation, satisfyingly closing the circle from where we began.

So, one idea that our recent findings raise is that metabolic changes implicated in cancer risk, or that occur during carcinogenesis, can temporarily bypass Knudson's two-hit requirement for *BRCA2*, provoking genome-wide mutation patterns implicated in carcinogenesis. ‘Metabolic bypass’ of Knudson's paradigm in this way might represent a widespread mechanism used by cancer cells to overcome intrinsic tumour suppressor mechanisms – and possibly, not just those executed by BRCA2. What I find particularly interesting is that elevated levels of aldehydes like methylglyoxal are well known to occur in patients with metabolic diseases like diabetes. Diabetes increases the risk of several cancers, including breast and pancreatic cancer, but it's not clear why. It is tempting to speculate that the ‘metabolic bypass’ mechanism we have discovered might be involved. Given that diabetes now affects over 500 million people worldwide – disproportionately many in Asia and developing countries – it is quite interesting to consider these new horizons in our approach to cancer prevention.

[…] as many as a third of all cancers are potentially preventable. The problem is that for a lot of the most common environmental influences […] the mechanisms underlying these connections remain elusive. […] until you understand these mechanisms better you cannot effectively create or implement new cancer prevention strategies.

**How do you think the field of cancer prevention is progressing?**

Cancer prevention has been around for a long time as a field, but I would say that, compared to mainstream research on cancer biology, it remains somewhat neglected. Of course, this is changing because people across the world increasingly recognise the health impact and economic value of delaying or preventing cancer in ageing populations. Increasing attention is also being paid to delaying or preventing relapse after primary therapy, which also has a major impact.

The statistic often quoted by people in the field is that as many as a third of all cancers are potentially preventable. The problem is that for a lot of the most common environmental influences, like diet and exercise, we have lots of lines of evidence suggesting that they contribute to our risk of cancer by intersecting with our genetic inheritance, but the mechanisms underlying these connections remain elusive. It's my proposition that until you understand these mechanisms better you cannot effectively create or implement new cancer prevention strategies. I think that our recent work on metabolism and tumour suppression helps to illustrate what I mean.

I've been fortunate enough to have had the opportunity – first in Cambridge, now in Singapore – to foster research along these lines. When I took over many years ago from Ron Laskey as the director of the MRC Cancer Unit, in Cambridge, I was able to formulate a then-distinctive mission for the Unit, towards understanding the earliest steps in the genesis of epithelial cancers, and using that knowledge to improve their early detection or therapy, and delay or prevent their onset. The MRC provided generous support to the Unit at a time when early intervention in cancer was not a mainstream endeavour, and it is satisfying to see these efforts perpetuated in Cambridge and elsewhere. Singapore well recognises the importance of early intervention to reduce the health and social impact of chronic diseases in their ageing population, and has instituted several initiatives to tackle this problem.

**What do you envisage being the most clinically impactful advance in cancer therapies in the near future?**

That's a difficult question, in answer to which I have no specific insights, and so I hope you will forgive me for segueing to my own work on technologies to accelerate drug discovery.

I started working in the area of chemical biology and early drug discovery about 10-15 years ago, motivated by a sense of frustration. It had long been clear that macromolecular complexes involving proteins are at the heart of cell regulation in physiology and in disease; yet, at that time, we had few available tools to target these complexes for drug discovery. My focus in this area has been on developing new technologies to help extend the druggable repertoire…by ‘drugging the undruggable’ so to speak. In this endeavour, my colleagues and I set ourselves a few key challenges.

The first challenge was to figure out how to identify the best novel drug targets in the macromolecular interaction space, in an efficient and unbiased way. To address this challenge, we developed a method we call ‘protein interference’. We first harnessed an extensive repertoire of small protein ‘shapes’ encoded in the genomes of simpler species, such as archaea and eubacteria, focusing on extremophilic species expected to have evolved structurally stable protein folds. We then introduced large, virally encoded libraries of these small protein shapes as probes into human cells, with the expectation that they would bind and interfere with the function of human cellular proteins, and thereby induce cellular phenotypes. This approach empowered rapid phenotypic screening for therapeutically relevant phenotypes, followed by isolation of the protein interference probe and their bound human targets, enabling rapid identification and assessment of the binding site. Somewhat to my surprise, the approach worked (Emery et al., 2021). Protein interference was spun out by Cambridge University into the start-up company, PhoreMost, which I co-founded with Chris Torrance and Grahame McKenzie. PhoreMost has since done a fantastic job using this technology to identify previously unknown druggable targets in collaboration with many major pharma companies, including Novartis, Bayer and Roche, as well as in its own internal drug discovery pipeline.

Macromolecular interactions are relatively inaccessible to approaches for ligand discovery, and so we faced the second challenge of how to drug them. Working with Chris Abell, who sadly passed away in 2020, Marko Hyvonen and Tom Blundell, we developed new structure-guided approaches in fragment-based drug discovery and established new chemical libraries, to expand the toolkit to ‘drug the undruggable’, with generous support from the Wellcome Trust and MRC. A series of papers the collaborative team has published over the past 5 years illustrates the potential value of these approaches (e.g. Narvaez et al., 2017; Scott et al., 2021; Stockwell et al., 2024).

We need to provide access to new technologies in low-income countries so they can work on problems that may not be at the forefront of commercial efforts in Europe or the United States, for example.

**You have co-founded several spin-out companies, as well as PhoreMost. How do you decide when a research project or technology is mature enough for this type of progression?**

Another difficult question! On reflection, I can think of a few ‘tipping points’ that may signify when something is mature enough to progress.

I've been more interested in developing commercially viable technology platforms or approaches that can accelerate drug discovery, in order to build a venture, rather than in simply developing a single drug asset to sell to the highest bidder. So, my upcoming points should be seen in that light.

The first tipping point obviously concerns the novelty, validity and applicability of what you have developed. Is it truly original? Has it been rigorously validated? Is there a clear and important application that can lead to commercially relevant outcomes? A portfolio of patents covering the underlying intellectual property rights is another vital requirement.

A second tipping point concerns the ability to ‘commoditise’ new technology platforms. By that I mean, in an academic setting, it's great if an experiment works absolutely right ten times, but for a commercially viable platform, it has to work right 10,000 times or a million times. Academic labs are not very well set up to commoditise, to standardise and to rigorously define approaches and procedures that can make a technology work over and over and over again.

A final tipping point concerns the breadth of applications. For example, with a technology like protein interference, you could potentially access therapeutic targets for everything from neurodegeneration to cancer. How can one academic lab work at that breadth? You can't. You can't raise the grant funding and you don't have the biological expertise. But a company can do these things in ways not accessible to academic labs.

But before I finish, let me dwell for a moment on another issue. Access to health care across the world is highly inequitable. On the one hand, in high-income countries, we spend tens of thousands of pounds to treat small groups of patients with the very latest drugs. On the other hand, in low-income countries, you have people dying of potentially treatable diseases for which there is no effective drug, in their tens of thousands, if not millions. There's no magic wand to level this playing field, but I do feel that access to drug discovery technologies has to become more equitable. We need to provide access to new technologies in low-income countries so they can work on problems that may not be at the forefront of commercial efforts in Europe or the United States, for example. That was one motivation for me to start the Centre for Chemical Biology and Therapeutics Development in Bangalore, India, with help over many years from the leadership of the Bangalore Life Sciences Cluster, and generous support from the Department of Biotechnology, Government of India. We recently had the wonderful news that the Bill and Melinda Gates Foundation will be working with us in Bangalore to help to develop new drugs for the health of the many rather than the few. I'm very excited about these new prospects.

**You recently became the Director of the Cancer Science at Institute of Singapore. How was the move and how is it shaping your research?**

I'm having the time of my life – great science, wonderful colleagues, and generous investment by the Singapore Government into biomedical research and its infrastructure! I've been very excited to move to Singapore after more than 30 years in Cambridge. Genetic ancestry affects the onset, progression and response to therapy of many diseases, including cancer. Over half of the world's population is of Asian genetic ancestry. Yet much of what we know about cancer comes from experimental models and patient samples that reflect European genetic ancestry. So, I believe that the Cancer Science Institute's mission to understand the pathogenesis of cancers prevalent in Asia, and use this new knowledge to improve clinical outcomes, is topical and important not only to Asia, but also to our Asian diaspora across the world.

Many common forms of cancer in Asian populations, like colon cancer, also occur elsewhere. Other forms of cancer occur at higher frequencies in Asia than in western countries, such as gastric or liver cancers, triple-negative breast cancer, or EGFR-mutant lung cancer in non-smokers. Finally, certain cancers occur almost exclusively in Asia or among Asians, like nasopharyngeal carcinoma. Together, these cancers present a set of scientific problems of unique importance to Asia that also expose underlying mechanisms of general significance.

So, a strong motivation behind my move has been to tap into Singapore's collective strengths in this area. Singapore's population is primarily of Chinese, Indian and Malay descent, reflecting large population groups across Asia. Singapore has very successfully focused on the connections between Asian genetic diversity and disease, with underpinning infrastructure (such as national databases for genomic information) to facilitate this research. In this environment, almost everybody knows one another, and so it's easy to find and talk to fellow scientists, leaders and policymakers in government, academia or industry. So, overall, it's been easy to quickly settle down after my relocation.

Over half of the world's population is of Asian genetic ancestry. Yet much of what we know about cancer comes from experimental models and patient samples that reflect European genetic ancestry.

**What do you enjoy doing outside of the lab and work?**

I have multiple interests outside of research, and I've been able to indulge a few of them after the move. I'm really interested in Asian culture, particularly through the lens of the great religions, like Hinduism, Buddhism, and others that have come from Asian and Eastern philosophy.

Before my move to Singapore, my experience was limited to India, Japan, and a few aspects of Korea and China. Now, it's easy to visit across Indochina, and witness the melding of cultures pushing eastward from India and westward from China. Seeing how that cultural fusion has led to so many wonderful creations, like Angkor Wat, has been one of the things I've enjoyed most after my move.

I also quite enjoy trekking and nature photography. I've been able to trek in the rain forests in Malaysia and Thailand. Plus, there are tropical beaches within a couple of hours drive in Malaysia, or an hour's flight away in Thailand. It's amazing! On the other hand, however, one of my favourite interests, fell walking in the English Lake District, is now harder to do. I guess my next few Wainwrights will just have to wait.
